# Relative replication capacity of phenotypic SIV variants during primary infections differs with route of inoculation

**DOI:** 10.1186/1742-4690-7-88

**Published:** 2010-10-13

**Authors:** Tasha Biesinger, Robert White, Monica T Yu Kimata, Brenda K Wilson, Jonathan S Allan, Jason T Kimata

**Affiliations:** 1 Department of Molecular Virology and Microbiology, Baylor College of Medicine, Houston, TX 77030, USA; 2 Department of Virology and Immunology, Southwest Foundation for Biomedical Research, San Antonio, TX 77227, USA; 3 Southwest National Primate Research Center, Southwest Foundation for Biomedical Research, San Antonio, TX 77227, USA

## Abstract

**Background:**

Previous studies of human and simian immunodeficiency virus (HIV and SIV) have demonstrated that adaptive mutations selected during the course of infection alter viral replicative fitness, persistence, and pathogenicity. What is unclear from those studies is the impact of transmission on the replication and pathogenicity of the founding virus population. Using the SIV-macaque model, we examined whether the route of infection would affect the establishment and replication of two SIVmne variants of distinct *in vitro *and *in vivo *biological characteristics. For these studies, we performed dual-virus inoculations of pig-tailed macaques via intrarectal or intravenous routes with SIVmneCl8, a miminally pathogenic virus, and SIVmne027, a highly pathogenic variant that replicates more robustly in CD4^+ ^T cells.

**Results:**

The data demonstrate that SIVmne027 is the dominant virus regardless of the route of infection, indicating that the capacity to replicate efficiently in CD4^+ ^T cells is important for fitness. Interestingly, in comparison to intravenous co-infection, intrarectal inoculation enabled greater relative replication of the less pathogenic virus, SIVmneCl8. Moreover, a higher level of SIVmneCl8 replication during primary infection of the intrarectally inoculated macaques was associated with lower overall plasma viral load and slower decline in CD4^+ ^T cells, even though SIVmne027 eventually became the dominant virus.

**Conclusions:**

These results suggest that the capacity to replicate in CD4^+ ^T cells is a significant determinant of SIV fitness and pathogenicity. Furthermore, the data also suggest that mucosal transmission may support early replication of phenotypically diverse variants, while slowing the rate of CD4^+ ^T cell decline during the initial stages of infection.

## Background

Human and simian immunodeficiency virus (HIV and SIV) undergo genetic and biological changes during the course of infection that correlate with increased viral load and disease progression. The evolution of the virus population results from direct competition of viral variants [1,2], intense immune pressure [3], and target cell availability [4,5]. Thus, viral fitness is a dynamic term and is dependent on the mutations and conditions under which viral replication is taking place. For example, CD8 epitope escape mutants may show increased fitness compared to wild type virus in context of a specific restricting HLA allele, but with a corresponding loss of replication capacity and, subsequently, lower levels of persistent replication [6,7]. These types of mutations may revert during transmission to an unrestricted-HLA recipient, indicating that they impair fitness *in vivo *[8,9]. Likewise, antiretroviral drug resistant mutants may show higher fitness compared to wild type virus in the presence of the inhibitor, but lower fitness when the drug is withdrawn [10-12]. Additionally, viral variants isolated during early and late stages of infection may differ in their phenotypic properties and pathogenicity, with late-stage variants demonstrating increases in replication capacity and virulence [13-16]. However, questions about HIV-1 fitness and pathogenicity have been incompletely addressed because of inadequate tissue culture assays and the absence of a suitable HIV-1 animal model of infection to confirm correlative observations by systematic examination of transmission and pathogenesis.

An alternative approach to address questions of HIV fitness and pathogenicity has been to use the simian immunodeficiency virus (SIV)-macaque model [17]. The advantage of the model is that the fitness and pathogenicity of a virus of known genotype and biological phenotype can be defined after experimental inoculation into multiple hosts. Studies have shown how immune pressure, by both the humoral and cellular immune responses of the macaque, affects replication and pathogenicity [18-22]. However, while cytotoxic T cell (CTL) escape mutations cause a loss of fitness, glycosylation changes in the envelope protein that reduce immunogenicity and neutralization enhance replication in the host. Other experiments have shown that enhancement of fitness and pathogenicity may involve more than selection due to immune pressure. SIV variants that evolve increased virulence compared to the parental virus have inherent gains in infectivity and replication capacity that result from mutations selected in various determinants within the viral genome, including, *env *gp41 [23], *nef *[22,24-31] and *gag ca *[32], *gag-pol *[33], and *rt *[34].

Earlier studies primarily focused on defining how HIV and SIV adapt to the environment of the host in order to persistently replicate. What is less clear from those studies is the effect of transmission and the properties of the infecting viruses on the replicative fitness and pathogenicity of the founding virus population, which is commonly different from variants present at later stages of infection and disease [17,35-37]. In the present study, we used the SIV-macaque model to examine whether the route of infection would affect the establishment and replication of two viral variants of distinct biological characteristics [22]. A comparison of dual-virus inoculation via intrarectal and intravenous routes demonstrates that a mucosal route of transmission allows greater relative replication capacity of a less pathogenic virus during co-infection with a more pathogenic virus, while limiting the rate of CD4^+ ^T cell decline during the early stages of infection. However, the variant that replicates more robustly in CD4^+ ^T cells eventually dominates. These results suggest that replication capacity in T cells is a significant determinant of SIV fitness, but that replication fitness of the dominant infecting virus may be reduced after mucosal transmission.

## Results

### Viral replication fitness in culture is host cell dependent

**Figure 1:**
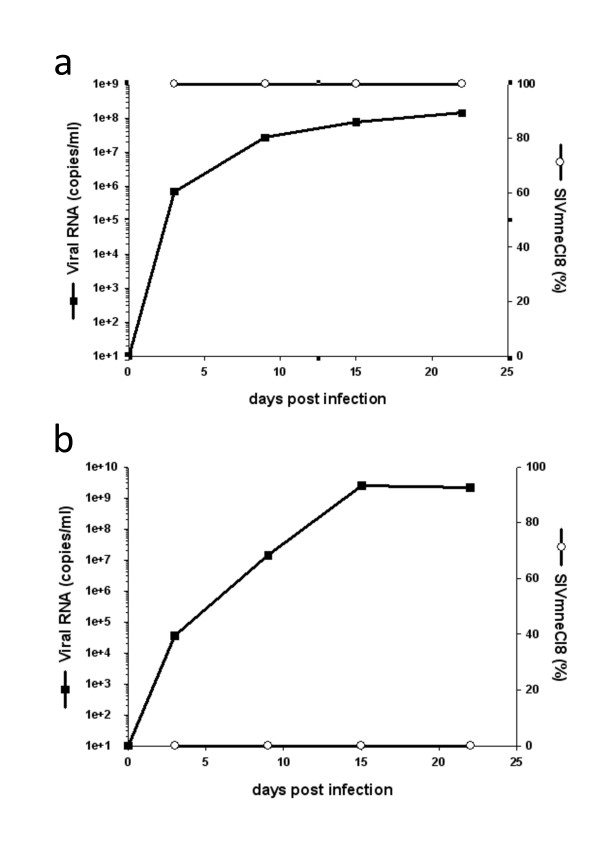
**Quantitative real-time RT-PCR to measure relative levels of SIVmneCl8 and SIVmne027**. The real-time RT-PCR assay was developed to detect two regions of the viral genome, a conserved *gag *sequence and an *env *V1 sequence specific to SIVmneCl8. The relative amount of SIVmneCl8 was determined from the amount of viral RNA detected by the SIVmneCl8 *env *V1 specific primer/probe set compared to the total amount of viral RNA detected by the primer/probe set recognizing the conserved *gag *sequence. Single-virus infections of activated pig-tail PBL with SIVmneCl8 (a) or SIVmne027 (b) shows that only SIVmneCl8 is recognized by the *env *V1 primer/probe, even when the overall viral RNA level is greater than 1 × 10^9 ^viral RNA copies/ml of SIVmne027.

*In vitro *competitive replication fitness assays have been used to determine the fitness of both HIV-1 and SIV [38-40], but whether these types of assays predict viral fitness and pathogenicity *in vivo *has not been examined. In order to address the predictive value of a cell culture assay for SIV fitness, we first determined the relative *in vitro *competitive replicative fitness of two CCR5-using phenotypic variants: a minimally pathogenic, parental, virus (SIVmneCl8) and highly pathogenic, late-stage variant virus (SIVmne027). Both viruses used in this study have been characterized during *in vitro *and *in vivo *single infection/inoculation studies (Table 1). Three types of pig-tailed macaque primary cell cultures were used to examine competitive replication: activated peripheral blood lymphocytes (PBLs), dendritic cell/T cell (DC/T cell) co-cultures, and monocyte-derived macrophage cultures. To monitor the relative amounts of SIVmneCl8 and SIVmne027 in cell-free culture supernatants over time, we developed quantitative real-time PCR assays that measure the overall amount of viral RNA by detection of a conserved SIV *gag *sequence and the amount of SIVmneCl8 by detection of a unique *env *sequence. Using known quantities of SIVmneCl8 and SIVmne027 viral RNA targets, we determined that the assay is capable of accurately quantifying SIVmneCl8 even in the presence of 10^5 ^to 10^6^-fold excess of SIVmne027 (data not shown). Furthermore, when activated pig-tail PBLs are infected with SIVmneCl8 alone, we observed a correlation between viral RNA measurements using the SIVmneCl8 *env *and SIV *gag *primer/probe sets (Figure 1a). As expected, SIVmneCl8 *env *was not detected in supernatants from SIVmne027 infected pig-tail (PBLs), even though there was about 3 × 10^9 ^viral RNA copies/ml of SIVmne027 as determined by total SIV RNA measurements (Figure 1b). This further indicated that there was insignificant detection of SIVmne027 by the SIVmneCl8 *env *specific real-time RT-PCR assay. Thus, the data show that SIVmneCl8 can be specifically detected by the allelic discriminating real-time PCR assay.

**Table 1:** Summary of the phenotypes of the SIV variants.

Virus Type	Name	*In vivo *pathogenesis		*In vitro *Replication Capacity					
		
		**Relative Viral Load**^**#**^	**CD4**^**+ **^**T cell decline**	Infectivity	Co-stimulated Lymphoblasts	Macrophages	Dendritic Cells	**Resting CD4**^**+ **^**T Cells**	DC-T Cell Co-cultures
Early	SIVmneCl8	1/1	Slow	Low	Low	Moderate	ND*	ND	Low

Late	SIVmne027	30/4000	Rapid	High	High	Low	ND	Low	High

^#^Differences in plasma viral loads during the acute and chronic stages (acute/set-point) following intravenous inoculation are indicated relative to SIVmneCl8.

*ND, not detected

References describing the variant viruses: [21,22,27,34,41,62-67]

**Figure 2:**
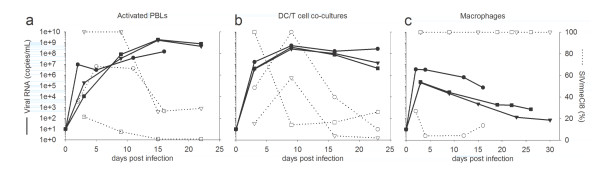
**Dual-virus competition assays in primary pig-tailed macaque cells**. Activated PBLs (a), DC/T cell co-cultures (b), and macrophages (c) were infected with equal doses of SIVmneCl8 and SIVmne027. Total virus was determined by measuring consensus sequence *gag *RNA transcripts (--). SIVmneCl8 viral RNA levels were measures and are reported as percentages of total viral RNA (····). Three representative experiments (black circle/open circle, black triangle/open triangle, and black square/open square) are shown for each graph and viral RNA values represent the average reading for each time point. Each infection used cells from a different macaque blood donor.

When we analyzed activated PBLs or DC/T cell co-cultures that were co-infected with equal infectious doses of SIVmneCl8 and SIVmne027, we observed that SIVmneCl8 represented a minor fraction (0-30%) of total viral RNA by the end of each experiment (Figure 2a and 2b). This occurred even if SIVmneCl8 represented greater than 90% of the virus at early time points after infection. Similar results were obtained with DC-T-cell capture-transfer assays (data not shown). By contrast, in two of three infections of monocyte-derived macrophages, SIVmneCl8 represented 100% of total viral RNA during co-infection (Figure 2c). These data correlate with the abilities of the two viruses to replicate under the three culture conditions in single-virus infections. For example, SIVmne027 replicates efficiently in activated PBLs and in DC/T cell co-cultures but to low levels in monocyte-derived macrophages. In contrast, SIVmneCl8 replicates to lower levels than SIVmne027 in activated PBLs and in DC/T cell co-cultures but to moderate levels in macrophages (Figure 1 and Table 1). Together, the data demonstrate that SIVmne027 has greater competitive replication fitness than SIVmneCl8 in activated PBLs and DC/T cell co-cultures, but not in macrophage cultures.

### SIVmne027 exhibits complete dominance over SIVmneCl8 following intravenous inoculation

**Figure 3:**
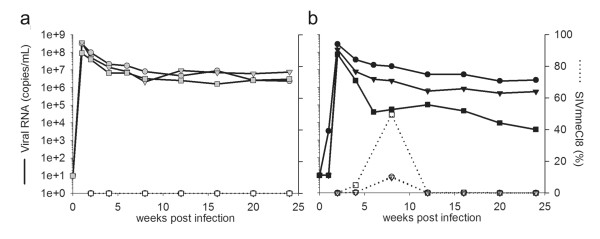
**Plasma viral loads from *in vivo *co-infections**. Macaques were infected with equal doses of SIVmneCl8 and SIVmne027 using either an intravenous route of inoculation (a), animals 29046 (gray circle/open circle), 29047 (gray triangle/open triangle) and 29048 (gray square/open square) or an intrarectal route of inoculation (b), animals 28488 (black circle/open circle), 28489 (black triangle/open triangle) and 28490 (black square/open square). Total virus in plasma was determined by measuring consensus sequence *gag *RNA transcripts (--) by quantitative real-time PCR. SIVmneCl8 specific viral RNA levels were also measured by quantitative real-time PCR and are reported as percentages of total viral RNA (····). Viral RNA values represent the average reading for each time point.

Since *in vitro *infection assays are limited to analysis of viral replication without immune pressures, we co-inoculated pig-tailed macaques with equal infectious doses of SIVmneCl8 and SIVmne027 intravenously (IV) in order to determine relative replicative fitness of these two viruses within a host. Overall virus replication in the three macaques peaked at week 1 post-inoculation between 10^8^-10^9 ^viral RNA copies/mL in plasma and stabilized around ~10^7 ^copies/mL (Figure 3a). Peak values and viral set-point levels in the co-infected animals are comparable to those seen during single-virus IV infections of pig-tailed macaques with SIVmne027 and about 30-1000-fold greater than infection with SIVmneCl8 [22,41]. SIVmneCl8 was undetectable at all times tested post-infection, indicating that it was unable to persist at a measurable level during IV co-infection with SIVmne027. The identification of a recombinant *env *sequence in animal 29046 provided confirmation of SIVmneCl8 infection in these animals (Table 2). These results show that SIVmne027 is dominant over SIVmneCl8 during IV co-infections. Furthermore, in context of earlier studies with these viruses [22], the data also suggest that variants that emerge with increased ability to replicate in CD4^+ ^T cells and which have increased pathogenicity are indeed more fit than viruses from which they evolved in the host.

**Table 2:** *Env *variants identified by single-genome cloning.

Source of DNA	***Env su *sequences**^**a**^	IR-infected Animals			IV-infected Animals		
		
		28488	28489	28490	29046	29047	29048
PBMC	SIVmneCl8	2	2	7	0	0	0
	
	SIVmne027	14	23	17	14	12	14
	
	Recombinant	0	0	0	1	0	0
	
	Total	16	25	24	15	12	14

^a^*Env su *fragments were cloned from PBMCs harvested eight weeks post infection by nested PCR amplification.

### SIVmneCl8 demonstrates higher relative replication levels after mucosal inoculation

**Figure 4:**
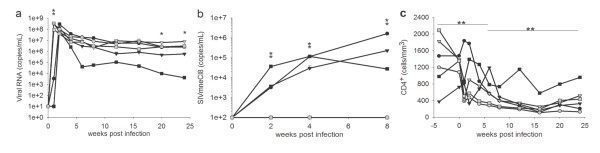
**Comparison of IV vs IR viral loads and CD4^+ ^T cell counts from SIVmneCl8/SIVmne027 co-inoculated macaques**. Total plasma viral RNA transcripts (a), SIVmneCl8 *env *transcripts (b), and CD4^+ ^T cell levels (c) from intravenously infected macaques (gray circles, triangles, and squares) and intrarectally infected macaques (black circles, triangles, and squares) are shown. Viral RNA measurements were determined as in Fig. 3. Values represent average readings for each time point ± the standard error. Levels of significance are denoted as follows: (**) p < 0.05, (*) p = 0.05. In panel (a) the p-value at peak plasma viral load is 0.0007. The p-value for differences in viral set-point at 20 and 24 weeks post-inoculation are 0.0544 and 0.0509, respectively. For panel (b) p-values at 2, 4, and 8 weeks post-inoculation are 0.049, 0.006, and 0.005, respectively. For panel (c) the p-value for period 0-8 weeks post-inoculation is 0.0013, and the p-value for period 8-24 weeks post-inoculation is 0.0006.

We next examined what effect a mucosal route of infection would have on relative viral replication in the host. Pig-tailed macaques were co-infected intrarectally (IR) with equal doses of the same SIVmneCl8 and SIVmne027 stocks used to inoculate animals IV. We again found overall viral peak values in plasma ranged between 10^8^-10^9 ^copies/mL (Figure 3b). Interestingly, post-acute plasma viral loads in IR-infected macaques varied considerably between individual macaques in comparison to those in IV-infected animals. Moreover, SIVmneCl8 was detectable throughout the acute stage of IR infection and peaked at 8 weeks post-infection. SIVmneCl8 plasma viral loads increased from undetectable to between 1 × 10^5 ^to 2 × 10^6 ^viral RNA copies/ml and represented between 10 and 50% of the virus population in plasma (Figure 3b and 4b). However, it eventually gave way to SIVmne027, which became completely dominant with time. Macaque 28490 had the highest levels of SIVmneCl8, which constituted as much as 50% of the total virus population at 8 weeks post-infection, and the lowest overall plasma viral load. The CD4^+ ^T cell population in animal 28490 was also well preserved compared to the other animals (Figure 4c).

### Mucosal transmission decreases viral load and rate of CD4^+ ^T cell decline

To determine whether the route of transmission affected overall viral load, CD4^+ ^T cell decline, and competitive replication fitness of SIVmneCl8 and SIVmne027, we compared the results from the IR and IV infections. Although there was no significant difference in the average peak plasma viral RNA measurements, we found peak viral replication significantly delayed (*p *= 0.0007) after IR inoculation. The viral set-point averages at weeks 20 and 24 were also reduced in IR-infected macaques as compared with IV-infected macaques but were marginally significant (*p *= 0.0544 and 0.0509, respectively) (Figure 4a). Secondly, a comparison of the level of SIVmneCl8 in plasma between IV and IR infected macaques revealed a significantly greater level of SIVmneCl8 at week 2, 4 and 8 (*p *= 0.049, 0.006 and 0.005, respectively) in IR-infected macaques, despite the presence of the more pathogenic variant, SIVmne027 (Figure 4b). This was verified by single-proviral cloning and sequencing of *env *from PBMC isolated at 8 weeks post-inoculation (Table 2).

**Figure 5:**
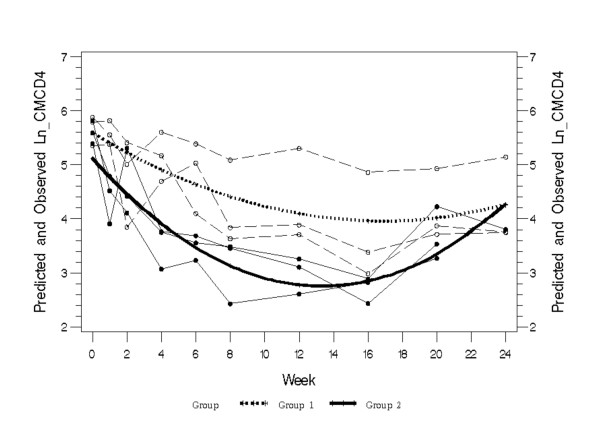
**Comparison of central memory CD4^+ ^T cells in intrarectally-infected and intravenously-infected pig-tailed macaques**. Individual data points from intrarectally-infected animals (thin dotted lines with open symbols) with the predicted mean value (thick dotted line; Group 1) were compared with data from intravenously-infected animals (thin solid lines with closed symbols) with the predicted mean value (thick solid line; Group 2). Significant differences were found at all time points from week 1 through week 20. P-values for all time points (0, 1, 2, 4, 6, 8, 12, 16, 20, 24) are given here: 0.0528, <0.0001, <0.0001, <0.0001, <0.0001, <0.0001, 0.0003, 0.0009, 0.0585 and 0.9761 respectively.

An analysis of absolute values of CD4^+ ^T cells showed a greater decrease in CD4^+ ^T cell levels in IV-infected macaques than IR-infected macaques (week 0-8: *p *= 0.0013; week 8-24: p = 0.0006) (Figure 4c). These differences were also reflected in the population of central memory CD4^+ ^T cells (Figure 5). Thus, although the more pathogenic and rapidly-replicating variant, SIVmne027, always dominated the co-infections, mucosal transmission enabled the slower-replicating SIVmneCl8 to replicate to relatively higher levels during the primary stage of infection. Interestingly, this was associated with lower viral loads and slower initial CD4^+ ^T cell decline, suggesting the induction of protective immune responses by SIVmneCl8. Taken together, the data indicate that there may be greater control of SIV after IR infection compared to IV infection.

## Discussion

We examined the relative replicative fitness of phenotypic SIV variants using both *in vitro *and *in vivo *co-infections in order to examine the impact of competition and target cell availability on relative viral replication and CD4^+ ^T cell decline during the early stages of infection. Our data demonstrate that a rapidly-replicating, highly pathogenic variant (SIVmne027) that evolved *in vivo *is indeed more fit than the slower-replicating parental virus (SIVmneCl8), regardless of whether co-inoculation was IV or IR. Furthermore, they confirm that *in vitro *competitive replication fitness experiments may predict replication fitness and pathogenicity *in vivo *[38-40]. The data support a model in which the predominant genotype established within a host is defined by how well a virus has adapted to replicating in CD4^+ ^T cells. They also demonstrate that these viruses are likely to predominate in subsequent transmissions. This model is consistent with previous *in situ *studies, which show that the primary target cells during transmission and acute HIV-1 and SIV infection are primarily CD4^+ ^T cells in lymphoid tissues [42-45].

Despite the dominance of the rapidly replicating variant, the slower replicating virus, SIVmneCl8, was able to expand exponentially during the acute stage after IR co-inoculation. It represented as much as 50% of the total virus population before giving way to SIVmne027. This was unexpected, given the nearly complete dominance of SIVmne027 that was observed after IV inoculation, the low competitive replication fitness of SIVmneCl8 in T cell and DC/T cell cocultures, and our previous observations that in single-virus infections, SIVmneCl8 demonstrates both lower initial spike and set-point plasma viral RNA copies/ml than SIVmne027 in pig-tailed macaques [22,41]. However, the peak levels achieved by SIVmneCl8 are within range of what has been observed when it is inoculated alone. One potential explanation is that the viruses target different cell types after IR inoculation. For example, the dominant virus, SIVmne027, may spread in abundant memory CD4^+ ^T cells. In contrast, the virus with greater relative replication fitness in macrophages, SIVmneCl8, may be mainly limited to replication in rectal macrophages, which are known to be susceptible target cells [14,46]. While challenging, it would be of interest to examine the infection profile of a dual-virus inoculation and the cell types harboring virus in the rectum and gut-associated lymphoid tissue during acute infection. Alternatively, increased levels of the slower replicating virus, SIVmneCl8, could be due to immunosuppression induced by the dominant T-cell tropic variant, SIVmne027. However, this seems unlikely since higher levels of SIVmneCl8 were associated with greater preservation of the CD4^+ ^T cell populations. Potentially, this association may indicate that SIVmneCl8 only replicates efficiently in a particular subpopulation of CD4^+ ^T-cells that are available after IR inoculation but are depleted in the IV infected animals.

Some earlier studies have shown that multiple HIV-1 variants can be transmitted in both men and women, followed by purifying selection [47,48]. On the other hand, more recent studies indicate that only single HIV-1 variants appear to be transmitted to new hosts from the index case [35,36,43]. Furthermore, a low dose SIV mucosal infection experiment with the SIV isolates SIVmac251 and SIVsmE660 also suggested single virus transmission was frequent [49]. However, a new study shows that multivariant transmission may occur more frequently in men having sex with men than during heterosexual transmission [50]. A second study demonstrates that multiple variants are commonly transmitted in low dose vaginal challenge of rhesus macaques with SIV [51]. The reasons for these contrasting results remain unclear. One explanation for the differences may be that bottlenecks in mucosal transmission appear to lessen only when transmission occurs with co-existing genital infections and inflammation [52]. Regardless, none of the studies distinguish between limited transmission of variants across the mucosal surface and competitive selection of variants during dissemination and establishment of infection as the cause for limited viral diversity.

The animals in this study did not have signs of anal inflammation. Also, they were not subjected to repeated low dose exposures to the SIV variants. Thus, we were not able to address the question of whether one virus is preferentially transmitted over the other. For the experiments, we used a minimal dose expected to allow 100% infection by both variants in order to address which variant was likely to establish a more robust persistent infection if both are given the opportunity to infect the animals. Our data agree with the potential for rapid purifying selection after transmission based on competitive replication fitness if more than one virus is transmitted [47,48]. The data also raise the possibility that one variant may be highly dominant even if multiple variants get transmitted. In this regard, caution should be taken in considering a vaccine design that only targets what appears to be a commonly transmitted single genotype of HIV-1. Other variants may simply be hidden because of the high replication fitness of the predominant virus or because they are controlled by the host immune response [53].

Our data suggest that the route of infection may have a significant effect on the level of viral replication in the host. Statistically significant lower plasma viral loads were observed following IR compared to IV inoculation. Thus, host-specific selective pressures exerted on the viral populations may play an important role in variant selection in addition to direct viral competition for resources. These results suggest that a mucosal route of infection exerts a modest selective pressure for viral variants with lower replicative fitness and pathogenicity compared to IV infection. Clearly, a shortcoming of our study is the limited number of animals used per group. Although the data are statistically significant, further experiments will be required to verify the results because of potential variability [54]. However, it is important to note that we have found in past experiments that significant differences are observable with limited numbers of pig-tailed macaques infected with SIVmne variant clones [22,41]. Moreover, our data are in agreement with previous studies with uncloned SIVmac251, which also showed decreased viral fitness, lower viral loads, and pathogenicity after intrarectal inoculation or passage by intravaginal inoculation of rhesus macaques [55,56]. Thus, unlike the gains in pathogenicity of SIV that occur with serial intravenous passage [22,25], vaginal and rectal routes of infection may not lead to increased virulence and rate of progression to AIDS. It is unknown if these findings are particular to the viruses used for the studies. The use of molecular clones will allow us to compare the results of the current experiments with those using a different combination of cloned variant viruses.

Previous studies of recombination in SIVmac-infected rhesus macaques indicated rapid selection for recombinants with increased replication fitness [57,58]. Recombination in the present study was rarely observed. One reason for the difference in results may be because the sequences analyzed in our study were primarily restricted to the *env *gene. However, we have also examined a limited number of complete proviral genomes (4 total) and *env-nef*-LTR sequences (approximately 40) PCR amplified from the IR-inoculated animals but have not found evidence for recombination (C. Gingaras and J.T. Kimata, unpublished observations). An alternative explanation may be a methodological difference. Both earlier studies on SIV recombination co-inoculated animals with two mutants with deletions in either *vpr/vpx *or *nef*. In that scenario, recombination may readily occur to form variants with wild type genomes that are more fit for persistent replication and are therefore dominant. In the current experiments, we co-inoculated two variants with different replicative and pathogenic phenotypes but no deletion mutations. Recombinants may not have had a clear advantage for persistence compared to the dominant SIVmne027 variant.

Whether the level of replication of the slower replicating SIVmneCl8 contributes to the decrease in plasma viral load that occurs after IR inoculation is unknown and will require further investigation. Although it is possible that SIVmneCl8 induces protective immune responses against SIVmne027, we previously demonstrated that a prime/boost vaccine based on SIVmneCl8 fails to protect or lower viral load in macaques challenged by another highly related variant SIVmne [41], suggesting the SIVmneCl8 infection may not prime an effective immune response against SIVmne027 either. However, we cannot rule out that a more robust immune response is induced during intrarectal infection preventing gains replic.

It will be of interest to determine whether continued mucosal passage of variants from SIVmneCl8/SIVmne027 infected macaques leads to additional reductions in viral fitness and lengthening of the time to disease, and to determine what immune responses contribute to the lower replication level of SIV after intrarectal inoculation. The results could provide important insights into host immune mechanisms that can contain infection and select for less pathogenic variants. As HIV-1 is primarily sexually transmitted, the reductions in viral load observed following mucosal infection and passage in the macaque may also provide an explain for why the overall rate of HIV disease progression in the general human population has remained relatively constant, or even decreased, despite the adaptations affecting pathogenicity that occur in the virus during the course of infection of an individual [59-61].

## Conclusions

Previous studies demonstrated that HIV/SIV variants that evolve during an infection have increased competitive replication fitness compared to the infecting virus. We further those studies by showing in the macaque host that an SIV variant with increased pathogenicity, dominates infection when co-inoculated either IR or IV into a host along with the parent virus from which it evolved. We conclude that replication fitness evolves with pathogenicity and that variants that replicate most efficiently in CD4^+ ^T cells are likely to dominate after subsequent infections. However, IR infection supported relatively higher replication of the less competitively fit virus despite replication of the more pathogenic variant, suggesting that the rectal environment provides greater target cell availability for replication of phenotypically diverse variants. Finally, our data agree with earlier studies suggesting that mucosal infections, unlike IV infections, may curtail increases in viral replication fitness and rate of CD4^+ ^T cell decline, thereby preventing an increase in the rate of disease progression with passage of the virus to new hosts.

## Materials and methods

### Cell culture and Viruses

Primary pig-tailed macaque cells were cultured in RPMI1640 supplemented with 10% heat-inactivated fetal bovine serum (HI-FBS), 2 mM L-glutamine, and 100 U/mL penicillin and 100 μg/mL streptomycin (P/S) (RPMI complete) with additional cytokines as required. sMAGI cells were maintained in DMEM, 10% HI-FBS, 2 mM L-glutamine, P/S (DMEM complete) with 0.20 mg/mL G418 and 50 U/mL hygromycin B. The cloning of the variant viruses, SIVmneCl8 and SIVmne027, were described previously [22,62,63]. Characteristics of each virus are summarized in Table 1[21,22,27,34,41,62-67]. Infectious SIVmneCl8 and SIVmne027 stocks were prepared by transfection of plasmid DNA containing the respective provirus into 293T cells, and virus stocks (infectious units (IU)/ml) were quantified using the sMAGI assay as described [68].

### *In vitro *viral infections

Peripheral blood mononuclear cells were isolated from pig-tailed macaque blood using Ficoll-hypaque isolation. Blood donor animals are different from those used for the *in vivo *inoculations described below. Monocytes were depleted by adherence and the remaining peripheral blood lymphocytes (10^5^-10^6 ^cells/well) were seeded into a 24-well plate coated with anti-CD3 and anti-CD28 per well for co-stimulation and then used for viral replication assays as described [34]. Equal infectious doses of SIVmneCl8 and SIVmne027 (MOI = 0.001 for each virus) were added to each well in ~200 μl volume and incubated for 3 hours. Cells were washed twice with PBS to remove any unbound virus and resuspended in RPMI complete + 50 U/mL IL-2 (RPMI/IL-2). Supernatant samples were collected and media replaced with RPMI/IL-2 every 2-3 days. Viral RNA was isolated using QIAamp Viral RNA minikit (Qiagen). Samples were analyzed with real-time RT PCR as described below to quantify total SIV and SIVmneCl8 specific viral RNA.

Dendritic cell (DC)/T-cell co-cultures were prepared from monocytes and T-cells isolated from the blood of individual macaques as described [67]. Five days after isolation, monocyte-derived DCs and T-cells were mixed and equal doses of both SIVmneCl8 and SIVmne027 (2 × 10^3 ^IU; MOI = 0.01 relative to T cells) were added to the co-cultures, incubated for 3 hours, and washed twice with PBS to remove unbound virus. Co-cultures were resuspended in RPMI/IL-2. Alternatively, viruses were first captured by DCs and then transferred to T-cells (DC-T-cell capture-transfer assay) as described [67]. Supernatant samples were collected and media replaced every 2-3 days. Viral RNA was isolated using QIAamp Viral RNA minikit (Qiagen). Samples were analyzed with real-time RT PCR as described.

Macrophages were differentiated from monocytes isolated by plastic adherence from pig-tailed macaque PBMCs as described previously [62,66]. Cultures were inoculated with equal doses of SIVmneCl8 and SIVmne027 (MOI 0.01), washed twice with PBS to removed unbound virus after 4 hours, and cultured in RPMI complete media. Supernatant samples were collected every 2-3 days and media replaced with fresh media. Viral RNA was isolated from samples using QIAamp Viral RNA minikit (Qiagen). Samples were analyzed with real-time RT PCR as described.

### *In vivo *inoculations of macaques

Six pig-tailed macaques (three per group) were infected either intravenously or by atraumatic intrarectal inoculation with 1 × 10^4 ^IU each of SIVmneCl8 and SIVmne027 using previously described methods [22,69,70]. This dose was experimentally determined to be the minimum required to inoculate 100% of pig-tailed macaques with an uncloned infectious stock of SIVmne by the rectal route [71]. Animal care and usage were performed in accordance with protocols approved by the Institutional Animal Care and Use Committee of the Southwest Foundation for Biomedical Research and were conducted in accordance with Animal Welfare Act guidelines.

### PCR cloning

*Env *fragments were cloned from DNA of PBMCs harvested eight weeks post infection by nested PCR using previously described methods [72]. Individual clones were amplified from PBMC DNA after limiting dilution of each specimen determined the minimal amount of DNA required to amplify a proviral *env *sequence. *Env *fragments were cloned into pCR2.1-TOPO vector using the TOPO TA Cloning Kit (Invitrogen) according to the manufacturer's protocol. Sequences were analyzed for identity to the parental viruses, SIVmneCl8 or SIVmne027, or for possible recombination.

### Real-time RT PCR Assay

RNA standards for detecting *gag *and SIVmneCl8 *env *V1 sequences were prepared from plasmid stocks pKS^+^-BamHI-KpaI (pKS^+ ^plasmid containing BamHI-KpaI fragment of SIVmneCl8 gag) and pSK^+^-Cl8.Env (pSK^+ ^plasmid containing the fragment 219-570 of SIVmneCl8 env) by *in vitro *transcription. RNA sample and standard dilutions were prepared in DEPC-H_2_O + 50 U/mL RNase Inhibitor (Invitrogen) and 0.1 μg/μL yeast tRNA (Sigma). Plates were prepared with 40 μL of an RT PCR master mix (25 μL 2× RT PCR master mix, 1.25 μL 40× RNase inhibitor mix, 12.92 μL DEPC-H_2_O and 0.83 μL 60× GAG primer/probe mix [forward primer: TGTCAGGGAAGAAAGCAGATGAATT; reverse primer: TGCCCATACTACATGCTTCAACAT; dye, probe sequence and quencher: FAM-CCGGGTCGTAGCCTAA-MGB NFQ] for *gag *detection and for specific detection of the SIVmneCl8 Env 12.5 μL DEPC-H_2_O plus [0.25 μL 200× forward primer CAACAGCACCAACAGCAATACC, 0.25 μL 200× reverse primer ACAAGGACTATTCTCATTGACCACTTT and 1.25 μL 40× Env Probe VIC-ACAAAAGCAGAGGCAAT-MGB NFQ] (Applied Biosystems). 10 μL of standard or sample was then added and plates were analyzed with a standard cycling procedure on a 7500 Real Time PCR System with SDS 1.0 software (Applied Biosystems). Non-templated and no RT controls were prepared as above, but substituting DEPC-H_2_O for template RNA or a 2× DNA Master Mix (Applied Biosystems) for the 2× RT PCR Master Mix, respectively. Validation assays were performed to demonstrate accuracy and specificity of the primer/probe sets.

### Flow Cytometry Analysis

CD4^+ ^T cell counts were determined by multiplying the total lymphocytes counts by the fractional amount of CD4^+^CD3^+ ^T cells. Central memory CD4^+ ^T cells were determined by multiplying the number of CD4^+ ^T cells by the fractional amount of CD95^+^CD28^+ ^cells in the CD4^+ ^T cell gate. Percentages of CD4^+ ^T cells were determined by 5-color flow cytometry using antibodies from Becton Dickinson: anti-CD3-Alexa 700 (SP34-2), anti-CD4-APC, anti-CD95-FITC (DX2), anti-CD8-PerCP, and anti-CD28-APC (28.2).

### Statistical Methods

For comparative analysis of total SIV virion RNA and that of SIVmneCl8 between IV and IR infected macaques a mixed model was used with fixed effects using SP (POW) for variance-covariance structure. This approach was also used for comparing memory CD4^+ ^T cells. To meet the normality assumption for the model, natural logarithm transferred variable (Ln_CMCD4) were used in the analysis. The analysis of total CD4^+ ^T cell counts used one-way ANOVA for repeated measures.

## Competing interests

The authors declare that they have no competing interests.

## Authors' contributions

JSA, JTK, and TB designed the experiments. TB performed most of the experiments, and analyzed the data. JSA, RW, and BKW assisted with the macaque infections and the flow cytometry analyses. MTY performed the PCR cloning. TB and JTK wrote the manuscript. All authors read and approved the final manuscript.
